# Viral persistence in colorectal cancer cells infected by Newcastle disease virus

**DOI:** 10.1186/1743-422X-11-91

**Published:** 2014-05-16

**Authors:** Suet-Lin Chia, Khatijah Yusoff, Norazizah Shafee

**Affiliations:** 1 Department of Microbiology, Faculty of Biotechnology and Biomolecular Sciences, Universiti Putra Malaysia, Serdang 43400, Malaysia; 2 Institute of Biosciences, Universiti Putra Malaysia, Serdang 43400, Malaysia

**Keywords:** Newcastle disease virus, Persistent infection, Colorectal cancer cell line

## Abstract

**Background:**

Newcastle disease virus (NDV), a single-stranded RNA virus of the family *Paramyxoviridae*, is a candidate virotherapy agent in cancer treatment. Promising responses were observed in clinical studies. Despite its high potential, the possibility of the virus to develop a persistent form of infection in cancer cells has not been investigated. Occurrence of persistent infection by NDV in cancer cells may cause the cells to be less susceptible to the virus killing. This would give rise to a population of cancer cells that remains viable and resistant to treatment.

**Results:**

During infection experiment in a series of colorectal cancer cell lines, we adventitiously observed a development of persistent infection by NDV in SW480 cells, but not in other cell lines tested. This cell population, designated as SW480P, showed resistancy towards NDV killing in a re-infection experiment. The SW480P cells retained NDV genome and produced virus progeny with reduced plaque forming ability.

**Conclusion:**

These observations showed that NDV could develop persistent infection in cancer cells and this factor needs to be taken into consideration when using NDV in clinical settings.

## Introduction

Newcastle disease virus is a negative-stranded RNA virus belonging to the family of *Paramyxoviridae* of the genus *Avulavirus*. Its high preference to infect cancer cells [1] have made it as one of the widely studied candidate agent for oncolytic virotherapies. Due to its promising potential, NDV strains NDV-HUJ, PV701, MTH-68/H and NV1020 are now being tested as cancer therapeutics in clinical trials [2-4]. Despite their promising preclinical findings [4], further advancement in the clinical use of NDV is still debatable even though their mechanistic insights are well studied [reviewed in 4]. The risks involved in using viruses as therapeutic agents include problems with mutant viruses having increased pathogenic properties [5] and potential development of persistent infections [6].

Viruses have the ability to establish a persistent infection in host cells. This type of infection may exist in the forms of silent or productive infections [6]. Cells, which have been persistently infected by viruses, remained viable in the forms of latent, chronic or slow infections [6]. The ability of enveloped negative strand RNA viruses such as arboviruses, paramyxoviruses, vesicular stomatitis virus (VSV) and rabies virus to develop persistent infections in host cells has been well documented [7-10]. The forms of ‘incomplete’ viruses [10-12] involved in these persistent infections were referred to as defective interfering particles (DIPs). For oncolytic viruses, development of persistent infections can lead to reduced virus-induced cytotoxicity [13]. It can also create populations of cells that have reduced permissiveness to wild-type viruses [10-13]. This phenomenon was previously reported for the oncolytic reovirus, where wild type reovirus cause reduced infection in persistently infected cells [14].

Development of persistent infections by NDV was first reported in the late 60s and early 70s [7-13]. These studies, however, only reported the occurrence of persistent infections in normal cells. Since NDV has shown great potential as an anticancer agent in clinical studies [2,3,15,16], detailed information regarding their involvement in persistent infection in cancer cells is imperative. Thus far, occurrence of persistent infection in cancer cells by a velogenic strain of NDV has not been described, even though its establishment in normal cells had been reported [8-12]. Therefore, in the present study, we reported the development of persistent infection by NDV in a subpopulation of SW480 colon cancer cells. These findings contribute additional data needed in the tailoring of NDV, particularly of a velogenic strain, as oncolytic virotherapy agent in clinical settings.

## Results

### Detection of a subpopulation of cancer cells that are resistant to NDV cytolysis

**Figure 1:**
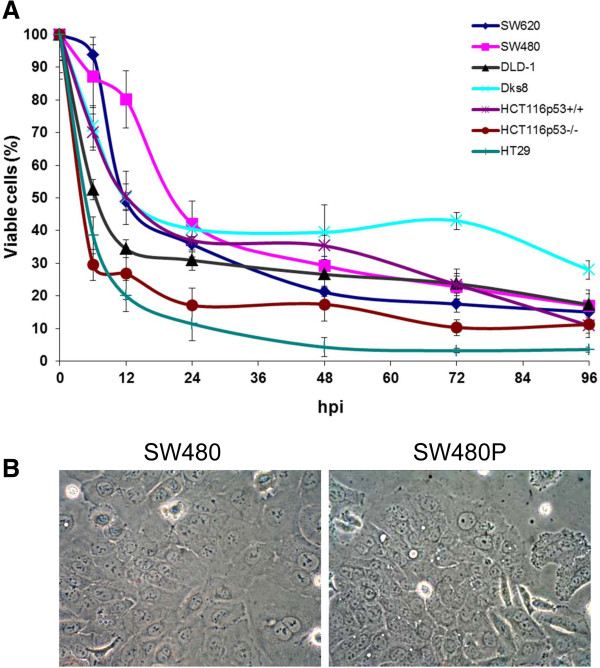
**Viability and morphology of CRC cancer cell lines following NDV infection. (A)** Viability of CRC cancer cell lines following selected times intervals post-infection with NDV strain AF2240. At 96 hours post-infection, only less than 40% cells remained viable across all the cell lines. **(B)** The remaining cells in the infected SW480 continued to proliferate following media replacement. These cells (SW480P) retained similar morphological features to the parental SW480 population.

During the course of our investigation into the oncolytic activities of NDV strain AF2240 in human colorectal cancer (CRC) cell lines, we inadvertently observed the existence of a subpopulation of SW480 cell line that was resistant to NDV killing. To characterize these resistant cancer cells, we attempted further culturing and propagation of the cells based on their continued proliferation and morphological observation. This population of cells originated from the remaining viable SW480 cells after 96 hpi (Figure 1A). Even though SW620, DLD-1, Dks8, HCT116p53^+/+^, HCT116p53^-/-^, and HT29 CRC cell lines also contained approximately 10-30% viable cells at this time post-infection, they died within 7 days following media replacement. The remaining cells in the infected SW480, on the other hand remained viable and they continued to proliferate following media replacement. The resulting population of cells was designated as SW480P. These cells retained similar morphological features to the parental SW480 population (Figure 1B). A complete confluency of the SW480P on flask surfaces was achieved within approximately 10 days after the media replacement.

### SW480P cells are less susceptible to NDV-induced cytolysis

**Figure 2:**
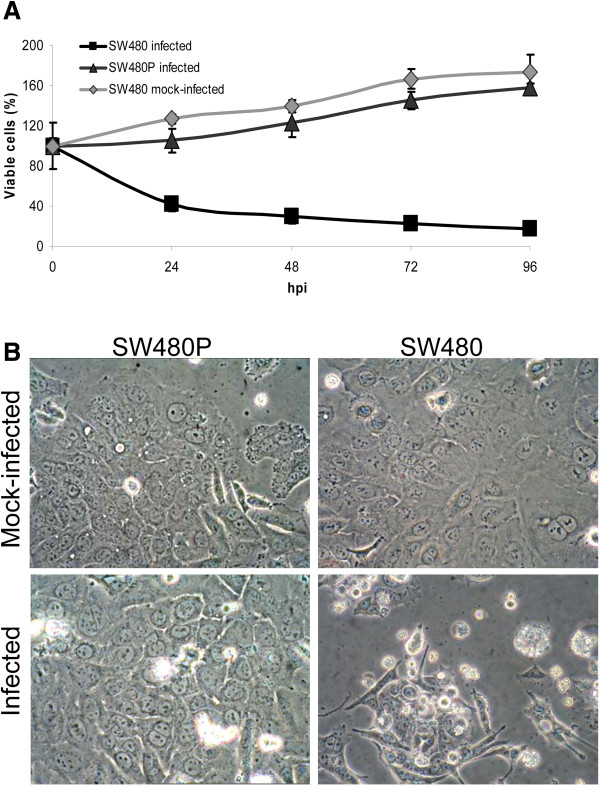
**Susceptibility of SW480P cells to NDV-induced cytolysis. (A)** Viability of re-infected SW480P cells compared to the parental SW480. The cells were re-infected with the similar MOI as the initial SW480 infection. **(B)** The infected SW480P cells did not show any obvious cytopathic effects (CPE) after 72 hpi while the parental SW480 displayed the typical pattern of CPE due to NDV infection.

The existence of a subpopulation of cancer cells that were resistant to NDV killing was suggestive of a persistent type of viral infection [6]. For oncolytic viruses, these persistently infected cells tend to be less susceptible to virus-induced cytolysis in subsequent infections [13]. To investigate whether this phenomenon occur in the SW480P, a re-infection studies were performed. Re-infection of the SW480P cells with the same multiplicity of infection (MOI) as the initial SW480 infection, did not result in cell death even after 96 hpi (Figure 2A). The cells instead continued to divide at almost the same multiplication rate as the mock-infected SW480 population. Even though a slightly slower replication rate was noted in the infected SW480P during the first 24 hours of infection, it stabilized to the level of SW480 afterwards. Infected SW480, on the other hand displayed the expected pattern of reduced cell viability as the infection period progressed. In addition to the absence of susceptibility to NDV killing, infected SW480P cells also failed to show any obvious cytopathic effects (CPE) after 72 hpi (Figure 2B, left panel). A parallel infection in the parental SW480, on the other hand, showed the typical pattern of CPE due to NDV infection (Figure 2B, right panel).

### NDV genes and proteins were detectable in the SW480P cells

**Figure 3:**
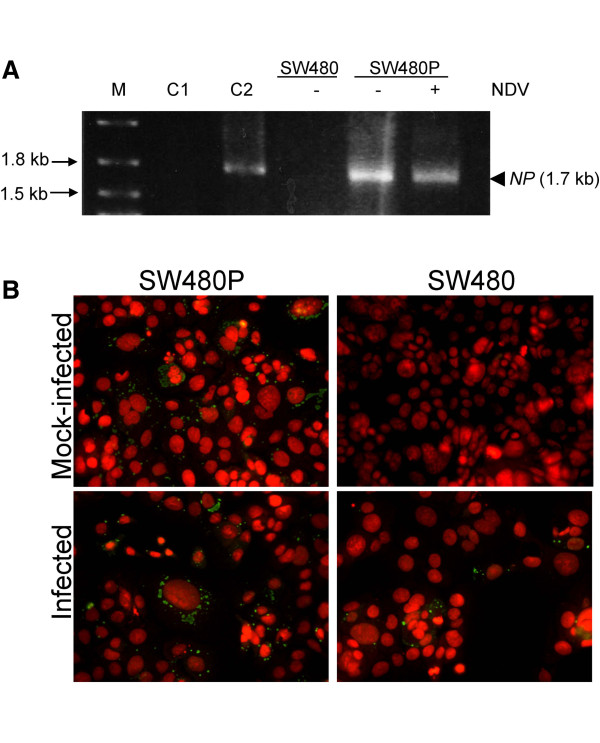
**Detection of NDV genes and proteins in the SW480P cells. (A)** RT-PCR analysis on DNA samples of SW480 and SW480P cells infected and mock infected with NDV. A DNA band with a size of 1.7 kb appeared in selected samples. **(B)** Immunofluorescent analyses of the infected and control cells using a monoclonal antibody against the NP protein of NDV. A speckled pattern of green antigen staining was visible in the cytoplasm of cells.

Resistance of the SW480P towards NDV-induced killing during the re-infection experiment was suggestive of a persistent NDV infection in the cells. Characteristics of persistently infected cells include reduced virus-induced cytotoxicity and a deregulation of antiviral response [17]. This was perhaps coincided with the presence of viral RNA and protein synthesis [11] within the hosts’ transcriptional and translational machineries. To investigate whether the SW480P cells retained NDV genes and proteins, we performed RT-PCR and immunofluorescent analyses as described in the Materials and methods. RT-PCR amplification showed a DNA band with a size of 1.7 kb, which was the expected size of an amplified nucleocapsid protein (*NP*) gene of NDV. This DNA band was seen in the positive control (Figure 3A, lane C2) and the SW480P samples (Figure 3A), regardless whether the cells were infected or not with NDV. On the other hand, no such band was seen in the negative control (Figure 3A, lane C1) and the mock-infected SW480 samples (Figure 3A).

Since DNA band of a similar size to the amplified *NP* gene was detected in the mock-infected SW480P cells, we were interested to see whether NDV proteins were also present in the samples. Immunofluorescent staining using a monoclonal antibody against the NP protein of NDV gave a positive detection in the mock-infected SW480P cells (Figure 3B, left panel). A speckled pattern of antigen staining (green) in the cytoplasm of cells, particularly in the perinuclear regions, was noted. Almost all the cells had this staining pattern. A positive staining for the NP protein was also seen in the infected SW480P as well as in the infected SW480 cells. No such staining was present in the mock-infected SW480 suggesting that the staining was specific for the NP protein of NDV.

### SW480P cells maintained a productive NDV infection, secreting virus progeny with reduced plaque-forming ability

**Figure 4:**
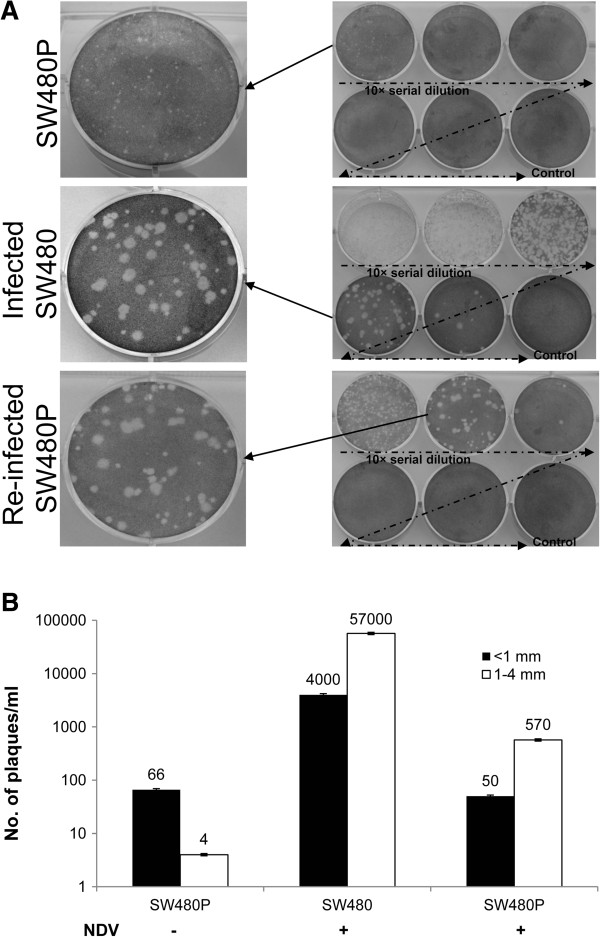
**Detection of infectious viral progenies secreted into cell culture media. (A)** A plaque assay was performed using spent culture media of the mock-infected SW480P and control cells. Different plaque morphologies were visible in the different samples. **(B)** A graphical representation of the number of plaques of less than 1 mm or in the range of 1-4 mm is shown.

Detection of NDV genome as well as proteins in the mock-infected SW480P suggested that the cells had been persistently infected with the virus, and were actively producing viral proteins. To further characterize the type of persistent infection [6] that occured in the cells, we determined whether any infectious viral progenies or just DIPs were being secreted by the cells. To achieve this, we performed a plaque assay [18] using spent culture media of the mock-infected SW480P cells. Results showed that plaques were formed (Figure 4A, top panel). However, their sizes were smaller than the plaques formed from the media of infected parental SW480 (Figure 4A, middle panel). These smaller plaque-forming virus progenies were designated as ‘mutant’ NDV (mNDV) particles. The majority of plaques from the mock-infected SW480P media had diameters of less than 1 (±0.05) mm while those from the infected SW480 media had a range of 1-4 mm. Media from the re-infected SW480P cells (after 96 hpi; Figure 4A, bottom panel), gave rise to a mixed morphology of plaques with diameters ranging from 0.5-4 mm. Upon closer examination, the number of small plaques (less than 1 mm) was lesser than the number of the bigger ones (1-4 mm) by a ratio of approximately 1:10.

Counting of the plaques showed that the undiluted spent media of the mock-infected SW480P had 66 pfu/ml of infectious virus progenies (Figure 4B), almost all of which were less than 1 mm in size. This was in contrast to the media from the infected parental SW480 and re-infected SW480P, where higher numbers of bigger plaques (1-4 mm) were seen. The number of total secreted infectious virus progeny was significantly higher in the infected parental SW480 cells compared to the SW480P cells. This was true even when the SW480P cells were re-infected with NDV.

### mNDV were infectious towards HT29 cells

**Figure 5:**
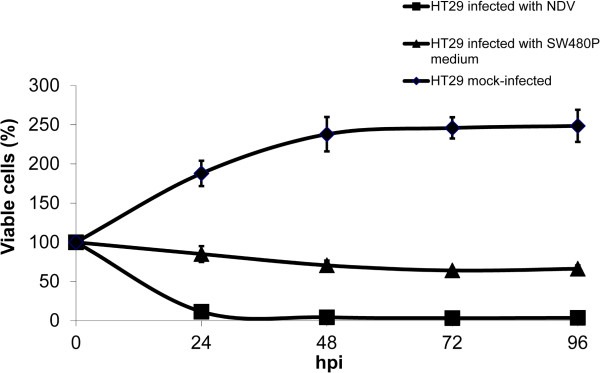
**Infectivity of mNDV on HT29 cells.** mNDV was infectious towards HT29 cells, however it resulted in less killing effect. The cells were infected using spent culture media from the mock-infected SW480P cells as described in the Materials and methods section.

Development of persistent infections was commonly associated with the production of ‘incomplete’ viruses termed DIPs [10-12]. DIPs are unable to sustain infection without the presence of suitable helper viruses. Plaque assay results in the present study showed that mNDV particles from the mock-infected SW480P culture media were able to infect and gave rise to plaques despite at smaller sizes. To investigate whether their infectivity on another colorectal cancer cell line was also retained, we performed infections in HT29 cells. HT29 cells were previously shown to be susceptible to NDV oncolytic activities [19]. Spent culture media collected from the mock-infected SW480P cells were used to infect HT29 as described in the Materials and methods section. After 24 hpi, viability of the infected HT29 cells remained around 90% (Figure 5). This value was different from the value of cells infected with the wild-type NDV, where only around 10% of cells were still viable. Higher percentage of viable HT29 cells was seen in the mNDV-infected culture compared to the wild type NDV-infected cells throughout the infection period. Even after 96 hpi, more than 70% of the mNDV-infected HT29 cells continued to be viable. This finding confirmed that mNDV remained infectious albeit at lower infectivity in HT29 CRC cells.

## Discussion

Development of persistent infection by oncolytic viruses may interfere with their efficacy as anticancer agents. This form of infection in target cells can affect wild type virus tropism as well as the cells permissiveness to reinfection [1]. It also caused a decrease in virus-induced cytotoxicity [13,20]. Data on the occurrence of persistent infections by a number of oncolytic viruses, such as Reovirus, Vesicular stomatis virus (VSV), measles, adenovirus and herpes simplex virus (HSV) were previously reported [20-24]. Up to now, there has been no report on the relationship of velogenic strain of NDV and persistent infection in cancer cells, despite the fact that the lentogenic strain of the virus is now in phases 1 and 2 clinical trials [4]. Development of persistent infection by NDV in normal cells, on the other hand, had also been well studied in normal cells [8-12], but not in cancer cells.

In the present study, we observed that a viscerotropic-velogenic strain of NDV [25] was able to develop persistent infection in a type of CRC cells, specifically the SW480 cell line. No such infection was observed in the other CRC cell lines tested. Further investigation into the contribution of genotypic differences among the cell lines towards NDV susceptibility and infection outcome are currently being investigated in our laboratory. The persistently-infected cells designated as SW480P, arose from a small subpopulation of cells which survived the primary round of NDV infection. SW480 cell line was also shown to be susceptible to cytolysis by other oncolytic viruses such as echoviruses [26]. This observation supports our data of the existence of persistently infected SW480 cells by NDV.

Persistent infection can be divided into latent, chronic, and slow infections [6]; each with its own unique characteristics that influence cellular changes. The lack of morphological changes in the SW480P versus SW480 cells, as well as the presence of viral NP proteins in almost all of the SW480P cells, were strongly suggestive of a slow type of persistent infection [6]. The absence of cell death, the occurrence of infectious virus secretion and the high fraction of antigen-positive cells in SW480P narrowed down the infection to a chronic diffuse type of persistent infection.

Previously, a plaque assay using media from persistently infected normal mouse cells on chicken embryonic fibroblasts also showed the formation of smaller plaques [10]. Even though the host cells were different in the assays, their data supported our observation that NDV can cause persistent infection. In the current study, we observed the occurence of a persistent infection in cancer cells. Such observation has not been previously reported in NDV infections.

These findings suggest that even though SW480P maintains a low level of productive NDV infection, they themselves are still susceptible to infection by the wild type NDV. On another note, the progeny virus, the mNDV, secreted by the SW480P cells retained their infectivity, hence they were unlikely to be in the form of DIPs per se. The DIPs are characterized by their inability to infect cells on their own due to large genetic mutations [27]. This mNDV virus was also able to infect another colorectal cancer cell line, HT29 [19] albeit at lower cytotoxicity. This suggested that the mNDV maintained its infectivity in other cancer cells besides the parental cells.

The fact that NDV was able to establish persistent infection in SW480 cells, but not in other cell lines tested, highlighted the specificity of either the mechanism of NDV infection in SW480 cells or the cells’ responses to the infection. To the best of our knowledge, until now only the lentogenic strains of NDV were evaluated in clinical studies [18]. This might be due to the specific regulations by the World Organization for Animal Health on the use of notifiable diseases and viruses with velogenic properties. Further investigation into the pathogenesis and oncolytic properties of the viscerotropic-velogenic strain of NDV, such as the one used in the study, would add to the understanding of their detail mechanistic actions. These data would contribute towards tailoring of velogenic NDV specificity in the clinical settings.

## Materials and methods

### NDV propagation

A viscerotropic-velogenic NDV strain AF2240 [25] was propagated in 9-day-old embryonated chicken eggs as previously described [28]. After 48 h of incubation, the infected allantoic fluid was harvested and clarified by centrifugation at 5000 × *g*. Virus was purified using a sucrose gradient centrifugation at concentrations of 20 to 60% at 200,000 × *g* for 4 h. A band observed around the middle of the gradient, which represented the concentrated virus, was pipetted and diluted with 1 × PBS (Sigma) followed by another centrifugation at 200,000 × *g* for 4 h. The resulting pure virus pellet was resuspended with 1 × PBS, aliquoted and kept in -80°C until used. NDV is endemic in Malaysia and categorized as a BSL2 pathogen based on NIH, US and Japan guidelines [29,30].

All experiments were performed in BSL2 biosafety cabinet. Extra precautions were taken to ensure there was not leakage of the virus to the environment. Waste and all instruments were sterilized and decontaminated after use.

### Cell lines and infection by NDV

CRC cell lines; SW620, SW480, DLD-1, Dks8, HCT116p53^+/+^, HCT116p53^-/-^, and HT29, were generous gifts from Prof. Eric J. Stanbridge, University of California, Irvine, USA. Cells were maintained in RPMI1640 media (PAA, Austria) supplemented with 10% (v/v) fetal bovine serum (FBS; PAA, Austria). For infection, cells were seeded for overnight followed by infection with NDV [18] at a 2.0 MOI. Cell viability was determined using the trypan blue exclusion assay. Experiments were repeated at least twice and a representative of the replicates was presented.

### Rescue of viable cells following NDV infection

After 96 h of infection, media containing floating cells were removed. Fresh growth media was added to the remaining attached cells. Cells were incubated for approximately two weeks with an addition of 4 ml of fresh growth media every 3 days. The surviving cells were then trypsinized and sub-cultured into new tissue culture flasks. For a re-infection experiment, the recovered cells were seeded and infected as described in the infection procedure above. Confirmation of NDV infection was performed using RT-PCR and immunofluorescent staining. Virus infectivity was quantitated using the plaque assay method [18].

### RT-PCR

Total RNA samples were harvested using the TriReagent (Invitrogen, USA) following the manufacturer’s protocols. First strand cDNA was then synthesized using the Reverse Transcription System (Promega) in a thermal cycler (MJ Research Inc. USA). Forward (5′- AAT GAA TTC TG ATG TCT TCC GTA TTC GAT G -3′) and reverse (5′-AAT CTC GAG C TCA ATA CCC CCA GTC GGT GT -3′) primers were used to amplify the NDV *NP* gene using 30 PCR cycles. The conditions were 94°C for 30 s, 55°C for 1 min, and 72°C for 1.5 min, followed by a final extension step of 72°C for 7 min. The resulting PCR product was electrophoresed, stained with ethidium bromide and viewed with a Gel Documentation Imaging System (BioRad, USA).

### Immunofluorescent detection of NDV NP protein

Infected cells on cover slips were washed with 1 × PBS and fixed with 4% paraformaldehyde, followed by permeabilization with 0.1% Triton-X 100 for 10 min. All dilutions of reagents were performed in PBS. After blocking with 1% BSA, cells were probed with a primary monoclonal antibody against the NP protein of NDV [31] for overnight. The bound antibody was then detected with an FITC-conjugated secondary antibody (Santa Cruz Biotechnology, sc-2010) and counterstained with propidium iodide. Samples were then visualized using a fluorescent microscope (DFC420C, Leica) and images were captured using a DM2500 (Leica) camera.

### Statistical analysis

Student’s *t*-test was used to analyze the experimental data throughout the study. Results were expressed as mean ± standard error of the mean of at least two independent experiments. Statistical significance was defined as p-value < 0.05. All the tests were performed using Windows Microsoft Excel 2010 (Microsoft Corporation, Seattle, WA).

## Competing interests

The authors declare that they have no competing interests.

## Authors’ contributions

NS, KY designed this study and revised the manuscript critically; SLC carried out this study and drafted the manuscript. All of the authors read and approved the final version of this manuscript.
